# Prevalence and correlates of persistent intracellular HIV transcription in individuals on efavirenz versus atazanavir-based regimens: A prospective cohort study

**DOI:** 10.1371/journal.pone.0194262

**Published:** 2018-03-13

**Authors:** Dimitrios Pilalas, Lemonia Skoura, Apostolia Margariti, Fani Chatzopoulou, Dimitrios Chatzidimitriou, Olga Tsachouridou, Pantelis Zebekakis, Simeon Metallidis, Maria Papaioannou

**Affiliations:** 1 1st Department of Internal Medicine, AHEPA University Hospital, Aristotle University Medical School, Thessaloniki, Greece; 2 National AIDS Reference Centre of Northern Greece, Department of Microbiology, Aristotle University Medical School, Thessaloniki, Greece; Katholieke Universiteit Leuven Rega Institute for Medical Research, BELGIUM

## Abstract

**Objectives:**

Despite successful virological suppression, HIV transcription frequently persists intracellularly. In this study, we hypothesize that HIV persistent transcription(HIVpt) may affect to a different extent patients on stable efavirenz(EFV) versus atazanavir(ATV)-based regimens. The role of the expression of drug efflux transporters in HIVpt was also investigated.

**Methods:**

We prospectively enrolled 51 virologically suppressed patients on first-line treatment for one year with EFV or ATV combined with emtricitabine and tenofovir and followed them up for one year. Simultaneous ultrasensitive subpopulation staining/hybridization in situ(SUSHI) was performed to identify HIVpt in CD4^+^ T-cells and in the CD4^+^CD45RO^+^ T-cell subpopulation. The differential mRNA expression of P-glycoprotein(P-gp/ABCB1) and multidrug resistance-associated protein-1(MRP1/ABCC1) was also evaluated. Univariate logistic regression models were used to evaluate predictors of HIVpt.

**Results:**

In the CD4^+^ T-cell population, HIVpt affected 13/30 of patients on EFV versus 10/21 on ATV. In the CD4^+^CD45RO^+^ T-cell population, HIVpt was present in 14/30 of patients on EFV versus 15/21 on ATV. A trend for association was observed between the risk of HIVpt and ATV treatment in the CD4^+^CD45RO^+^ T-cell population (OR 2.86, 95% CI 0.87–9.37, p = 0.083). HIVpt status was not associated with loss of virological suppression or CD4 evolution. We found no evidence of differential expression of the drug efflux transporters P-gp and MRP1.

**Conclusions:**

Further study is required to evaluate whether the HIVpt profile in specific cell populations may differ across different antiretroviral regimens and to elucidate the potential clinical impact.

## Introduction

Combination antiretroviral therapy(cART), albeit not curative, has improved substantially the morbidity and mortality of HIV disease through prolonged and sustained suppression of viral replication.[1] The establishment of HIV latency in a population of long lived memory CD4^+^ T cells is perceived as the major barrier for the eradication of HIV infection.[2] Several lines of evidence support the notion of persistent low level viral replication on cART at least in a subgroup of patients.[3–6] The importance of persistent HIV transcription(HIVpt) in patients on suppressive ART is unclear, but several studies suggest that biomarkers of HIVpt may be promising to assess residual viral replication.[7]

There is limited evidence derived from raltegravir intensification studies that residual viral replication as evidenced by increases in 2-long terminal repeat circles may affect to a lesser extent patients on non-nucleoside transcriptase inhibitors(NNRTIs) versus protease inhibitors(PIs).[3,4] For drugs with steep dose-response curves such as PIs, small decreases in intracellular concentrations may diminish significantly the viral inhibition providing a plausible explanation for this observation.[8] Antiretroviral drugs may be substrates, inhibitors or inducers of ATP-binding cassette transporters(ABC transporters) which function as drug efflux pumps with possible implications for the intracellular concentrations of antiretrovirals.[9]

In our prospective study, we used a method which combines immunophenotyping with ultrasensitive-FISH to detect unspliced HIV-1 gag-pol in relevant cell populations in order to compare HIVpt in virologically suppressed patients on efavirenz(EFV) or atazanavir/ritonavir(ATV/r) and a backbone of emtricitabine-tenofovir(FTC-TDF).[7,10,11] We followed prospectively our patient cohort for one year and investigated the impact of HIVpt on virological outcomes and CD4+ T-cell recovery. Finally, we tested for differences in the mRNA expression of two widely studied ABC transporters (P-glycoprotein, P-gp/ABCB1 and multidrug resistance-associated protein-1, MRP1/ABCC1) in peripheral blood mononuclear cells(PBMCs) between patients with and without HIVpt by treatment regimen.

## Patients and methods

### Study population and design

We conducted an observational prospective cohort study enrolling HIV-1 infected patients followed in the HIV clinic of the AHEPA University Hospital in Greece. The study was approved by the University Bioethics Committee and all patients provided written informed consent prior to study inclusion. Eligible patients were aviremic HIV-1 infected adults without hepatitis B or C co-infection on stable treatment for 12 months with either efavirenz or atazanavir/ritonavir and a backbone of emtricitabine—tenofovir disoproxil fumarate. Patients should be virologically suppressed (<50 copies/ml) as determined with the standard of care method (Roche COBAS TaqMan HIV-test, v 2.0) at sampling and for the preceding 6 months. Age, risk factor for HIV diagnosis, country of origin, pretreatment viral load, nadir CD4 count, CD4 count within 3 months before enrollment (baseline CD4 count), CDC stage as well as time from HIV diagnosis to treatment initiation and time from treatment initiation to study enrollment were recorded. Patients were followed up according to guideline recommendations for clinical progression and virological suppression for a period of one year after sampling. The CD4 count one year after sampling was recorded to evaluate the impact of HIVpt on CD4 evolution. In a post hoc calculation, a sample of 22 patients per treatment group would have 80% power to detect a 40% difference in the prevalence of HIVpt between the two treatment groups (α = 0.05).

### Samples

We isolated PBMCs from 6ml of whole blood using Histopaque(Sigma Aldrich, USA), within 4 hours after collection. Viability and cell count were performed by using 7-AAD (Immunostep, Spain) staining. An average number of 3.96 million cells from each individual were stored in -20 °C for the total RNA extraction.

### Simultaneous ultrasensitive subpopulation staining/hybridization in situ(SUSHI)

In our study, we focused on HIVpt in the CD4^+^ as well as in the CD4^+^CD45RO^+^ memory T-cell subpopulation. Cell samples were incubated with appropriate volumes of CD4-PE and CD45RO-PE-Cy5 monoclonal antibodies (Biolegend, USA) and the expression of intracellular HIV-1 unspliced gag-pol HIV RNA was evaluated with SUSHI (ViroTect, IncellDx, USA) according to manufacturer’s instructions. [12] Six HIV uninfected volunteers provided whole-blood samples and used as controls. We tested samples from 6 healthy volunteers with SUSHI and the percentage of positive cells ranged from 0.10% to 0.23%(median 0.16%). We adopted a cutoff of 0.3% which has been shown to correspond to 10–20 copies of HIV gag-pol unspliced RNA per cell and has been associated with significant lymphoproliferative responses to HIV-1 p24 in vitro. [10,13] Measurements were conducted in a CyFlow Space flow cytometer(Sysmex-Partec).

### Real-time qPCR analysis

Total RNA was extracted from PBMCs using the RNeasy Mini Kit for application in QIAcube automated system, according to the manufacturer’s instructions(Qiagen). RNA concentration was determined with the NanoDrop 1000(Thermo Scientific); in order to evaluate the integrity and quality of the isolated RNAs, random samples were checked using an Agilent 2100 Bioanalyzer. Extracted RNA was stored in -80 °C. Total RNA (500 ng) was reverse-transcribed using RT^2^ First Strand Kit(Qiagen), according to the manufacturer’s instructions.

We performed qPCR using Custom RT^2^ Profiler PCR Array(Qiagen) to compare the mRNA expression levels of P-gp(ABCB1) and MRP1(ABCC1) in patients with and without HIVpt on EFV-based versus ATV-based regimens. cDNA was combined with 2x RT^2^ SYBR Green MasterMix(Qiagen), and loaded onto an optically clear 96-well plate in duplicate where gene specific primers for target genes (ABCB1, NM_000927; ABCC1, NM_004996) and endogenous control gene (GSUB, NM_000181) were preloaded. At the same plate, genomic DNA contamination, efficiency of the reverse transcription reaction and efficiency of the polymerase chain reaction were tested by amplification of the genomic DNA control (HGDC, SA_00105), the reverse-transcription control (RTC, SA_00104) and positive PCR control (PPC, SA_00103) respectively. The cycling conditions were as follows: 95°C for 10 min, (95°C for 15 sec, 60°C for 1 min) × 40 cycles on the 7500 Fast Real-Time PCR System (Applied Biosystems).

### Statistical analyses

Continuous variables were summarized as mean and standard deviation(SD) for normally distributed data and as median and interquartile(IQR) otherwise. Discrete variables were expressed as percentages and 95% exact binomial confidence intervals(CI) were calculated. The independent samples t-test was used to compare normally distributed continuous variables and the Mann–Whitney for non-normally distributed variables. The chi-square test and the Fisher’s exact test were used for discrete variables as appropriate. The association of various predictors with persistent HIV transcription status was evaluated with univariate logistic regression models. All statistical tests were two-sided. P-values less than 0.05 were considered statistically significant.

For gene expression analysis, raw Cq values of the expression of control and target genes were exported. The relative expression levels were calculated with the ΔΔCq method and fold-changes as 2^-ΔΔCq^. The expression levels were analyzed with Student’s test and a sensitivity analysis was conducted with one-way ANOVA.

Statistical analyses were performed with IBM SPSS Statistics version 23 except for the sample size and the binomial confidence intervals calculations (R version 3.3.1 –stats(power.prop.test) and binom packages).[14,15] Data visualization was executed with GraphPad Prism version 7.0(GraphPad Software).

## Results

During the period from October 2012 to July 2014, a total of 51 participants were enrolled in the study (30 on EFV and 21 on ATV). The two groups were similar in baseline cohort characteristics with the exception of time on HAART, which was longer in the ATV group (36 vs 27 months, p = 0.044) (Table 1).

**Table 1:** Cohort characteristics.

	Overall (N = 51)	EFV group (N = 30)	ATV group (N = 21)	p-value
Gender n, (%)				0.391
Male	46 (90.2%)	26 (86.7%)	20 (95.2%)	
Female	5 (9.8%)	4 (13.3%)	1 (4.8%)	
Foreign origin n,(%)	3 (5.9%)	2 (6.7%)	1 (4.8%)	0.999
Risk factor for HIV infection				0.872
MSM	38 (74.5%)	23 (76.7%)	15 (71.4%)	
Heterosexual contact	11 (21.6%)	6 (20%)	5 (23.8%)	
IDU	2 (3.9%)	1 (3.3%)	1 (4.8%)	
Mean age (SD), years	42.2 (11.8)	40.1 (9.1)	45.2 (14.5)	0.123
CDC stage at diagnosis n (%)				0.575
A	33 (64.7%)	21 (70%)	12 (57.1%)	
B	15 (29.4%)	8 (26.7%)	7 (33.3%)	
C	3 (5.9%)	1 (3.3%)	2 (9.5%)	
Months from HIV-1 diagnosis to ART(IQR)	20 (5–49)	26 (7–57)	18 (4–44)	0.117
Months on ART (IQR)	31 (20–41)	27 (19–33)	36 (21–48)	0.044
Nadir CD4^+^ cell count cells/mm^3^,median (IQR)	294 (191–331)	301(229–330)	223(153–349)	0.284
Baseline CD4^+^ cell count cells/mm^3^, median (IQR)	613 (480–786)	606 (527–702)	687 (444–809)	0.950
Pre ART viral load (copies/ml) log_10,_ SD	4.81 (0.69)	4.72 (0.74)	4.94 (0.61)	0.261

EFV: on treatment with efavirenz, tenofovir, emtricitabine, ATV: on treatment with atazanavir, tenofovir,emtricitabine, MSM: men having sex with men, IDU:intravenous drug users, SD: standard deviation, IQR:interquartile range, ART: antiretroviral treatment

**Fig 1:**
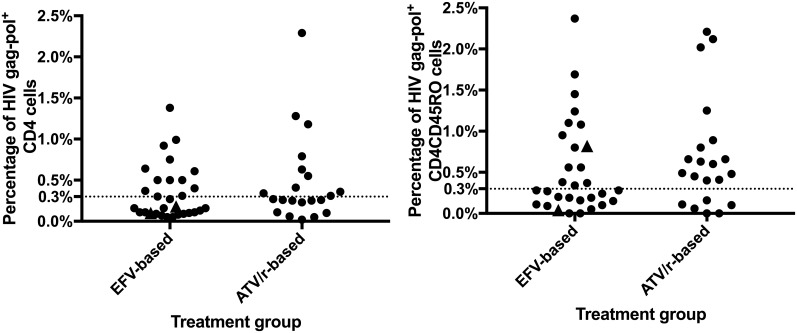
Simultaneous ultrasensitive subpopulation staining/hybridization in situ (SUSHI) patient results by antiretroviral regimen in CD4^+^ and CD4^+^CD45RO^+^ T-cells. Two patients experienced a transient increase of their viral load in the follow-up period (black triangles). EFV: efavirenz, ATV/r: atazanavir/ritonavir.

Persistent unspliced HIV RNA transcription as captured by SUSHI was identified in the CD4^+^ cell populations in 23/51 patients (45.1%, 95% CI 31.1 to 59.7%): 13/30 patients in the EFV group(43.3%, 95% CI 25.5 to 62.6%) versus 10/21 in the ATV group(47.6%, 95% CI 25.7 to 70.2%). In the CD4^+^CD45RO^+^ cell population, persistent HIV transcription was present in 29/51 patients (56.9%, 95% CI 42.2 to 70.7%) with 14/30(46.7%, 95% CI 28.3 to 65.7%) in the EFV group versus 15/21 in the ATV group (71.4%, 95% CI 47.8 to 88.7%). The percentage of cells with persistent HIV transcription for each patient sample is depicted in Fig 1. In patients with HIVpt the percentages of HIV gag-pol^+^ cells were not different between the two treatment groups (p = 0.693 for CD4^+^ cells and p = 0.747 for CD4^+^CD45RO^+^ cells).

In univariate logistic regression analysis, we observed a trend between the risk of HIVpt in the CD4^+^CD45RO^+^ cell population and ATV treatment (OR 2.86, 95% CI 0.87–9.37, p = 0.083) (Table 2).

**Table 2:** Predictors of HIV persistent transcription(HIVpt) in univariate logistic regression models.

Predictor	HIVpt in CD4^+^ cells OR (95% CI)	p-value	HIVpt in CD4^+^CD45RO^+^ cells OR (95% CI)	p-value
ATV regimen (reference EFV regimen)	1.19 (0.39–3.64)	0.762	2.86 (0.87–9.37)	0.083
Age per 10year increase	1.41 (0.85–2.31)	0.181	1.49 (0.89–2.50)	0.133
Nadir CD4 count per 100 cells/mm^3^ increase	0.96 (0.66–1.41)	0.835	1.02 (0.69–1.49)	0.933
CD4 count at sampling per 100 increase	0.85 (0.66–1.09)	0.191	0.96 (0.77–1.21)	0.742
Pre ART viral load (copies/ml) per 1 log_10_ increase	1.25 (0.55–2.81)	0.596	1.19 (0.53–2.67)	0.682

ATV: on treatment with atazanavir, tenofovir, emtricitabine, EFV: on treatment with efavirenz, tenofovir, emtricitabine, ART: antiretroviral treatment, OR: odds ratio, CI: confidence interval

After study enrollment and sampling, one patient from the ATV group was lost to follow-up and one patient from the EFV group had a treatment switch due to renal toxicity to tenofovir. No patient presented clinical progression. Viral loads were below the limit of detection throughout the observation period in all patients, except for two patients in the EFV group who presented transient viral load elevations. Viral load was below the level of detection in subsequent evaluations. Of note, both patients were classified as negative with regard to HIVpt in the CD4+ T cell population and one was classified as positive in the CD4^+^CD45RO^+^ cell reservoir (Fig 1). A mean increase of 47 cells/ml (95 CI% 10 to 84 cells/mm^3^) was observed with no evidence of a difference between the two treatment groups(p = 0.240). HIVpt status in CD4^+^and CD4^+^CD45RO^+^ cells was not associated with the evolution of the CD4 cell count (p = 0.945 and p = 0.962, respectively-data available for 40 patients).

We did not observe differences in the levels of mRNA expression of P-gp and MRP1 between patients in the two treatment groups (P-gp: 1.14-fold difference, 95% CI 0.88 to 1.47 and MRP1: 1.05 95% CI 0.91 to 1.2). Furthermore, there was no evidence for differential expression of P-gp and MRP1 in patients with HIVpt versus those without HIVpt overall as well as in the two treatment subgroups.

## Discussion

In our prospective study, we compared the prevalence of HIVpt as evaluated by SUSHI among virologically suppressed patients on two popular first-line regimens at the time the study was designed (EFV-based versus an ATV-based). HIVpt status was confirmed in approximately half of the patients. There was no evidence of a difference in the prevalence of HIVpt between the EFV-group and the ATV-group in CD4^+^ cells. However, a trend for an association between an ATV regimen and the risk of HIVpt was observed in the CD4^+^CD45RO^+^ cell population. Furthermore, we did not observe an association between HIVpt status and CD4^+^ count evolution and the two patients with transient elevations of viral load that were observed did not map consistently into the HIVpt positive population. Finally, our data do not support a differential expression of the drug efflux transporters P-gp and MRP1 in PBMCs of patients with and without HIVpt.

The shift of the HIV research agenda towards strategies for the quantification and eradication of the viral reservoir has renewed interest in biomarkers beyond CD4^+^ cell counts and plasma viral load.[16] Cell-associated viral RNA(CA-RNA) in PBMCs has been linked to decreasing CD4+ T cell counts in untreated HIV patients,[17] and suggested as a predictor of treatment failure in patients on HAART.[18] Chronic immune activation and dysfunction seem to correlate with cell associated measures of viral persistence but further research is needed to disentangle the interactions between them.[19–22]

We used a conceptually different method which identifies individual cells with low levels of HIVpt, instead of relying on average values that may be skewed importantly by a minority of cells with high levels of CA-RNA. SUSHI has been used previously to evaluate the “active” reservoir of HIV which was linked to persistent HIV-1 specific immune response.[10,12] The prevalence of HIVpt in virologically suppressed patients in our study is in line with previously published data.[10] In a recent study, similar levels of CA-RNA were found in PBMCs of PI versus NNRTI-treated aviremic patients,[20] and we replicated this finding with SUSHI in CD4 cells. However, different HIVpt profiles may be found in specific cell populations such as the memory CD4^+^CD45RO^+^ cells. Despite the fact that patients in the ATV group were on HAART for a longer period compared to the EFV group (36 vs 27 months, p = 0.044), the decay dynamics of cells with persistent HIV transcription suggest that such a difference should not impact the results meaningfully. [23]

Previous studies have suggested a role for CA-RNA as a predictive biomarker of virological failure(VF).[18] Our study was underpowered to evaluate such an outcome, but we were able to calculate the upper limit of the 95% confidence interval for the risk of virological failure in patients with HIVpt to 12.2%(0 VF cases out of 23 cases with HIVpt).[24] Furthermore, associations of HIVpt with pretreatment viral load and CD4 counts at sampling or before treatment initiation that have been previously reported were not replicated in our study.[18,20] Finally, HIVpt status was not associated with decreased in CD4 cell recovery one year after sampling.

P-glycoprotein and MRP1 are among the most studied drug transporters that affect the disposition of antiretroviral drugs. We hypothesized that the interactions of antiretroviral drugs with these molecules may reduce the intracellular drug levels and be associated with HIVpt in PBMCs. Similarly, increases in CA-RNA levels in the absence of virological failure have been described in patients with suboptimal adherence to cART. [25] The variation in the mRNA expression of these transporters was not associated with virological failure or CD4 recovery in a small prospective study.[26] Our findings also do not support a correlation between drug transporters and HIVpt. Nevertheless, it should be noted that the interactions between antiretroviral drugs and the transporters are quite complex. [27]

Several limitations apply to our study. The identification of HIVpt with SUSHI may not reflect concomitant HIV protein production as transcripts may be defective. Nevertheless, such transcripts may still encode chimeric proteins with implications for HIV pathogenesis.[22] Except for the total CD4^+^ cells, we examined the CD4^+^CD45RO^+^ memory cell population which includes cells that contribute significantly to the latent HIV reservoir such as the central memory and effector memory cells.[28,29] However, other relevant populations such as the memory stem cell population were not taken into consideration.[28] Similarly, our mRNA expression analysis was performed in total PBMCs and the transporter expression profile may be different in cell subpopulations. Further analyses of transporter protein expression with simultaneous determination of intracellular drug concentrations may provide further insights.

Large prospective studies with representative patient samples are required to validate the use of new biomarkers against clinical outcomes. Further study is required to evaluate whether persistence of HIV transcription may not affect to the same extent different cell populations across treatment regimens.

## References

[1] Wandeler G, Johnson LF, Egger M. Trends in life expectancy of HIV-positive adults on antiretroviral therapy across the globe: comparisons with general population. Curr Opin HIV AIDS 2016;11:492–500. doi: 10.1097/COH.0000000000000298 27254748 10.1097/COH.0000000000000298 PMC5055447

[2] Siliciano JD, Kajdas J, Finzi D, Quinn TC, Chadwick K, Margolick JB, et al Long-term follow-up studies confirm the stability of the latent reservoir for HIV-1 in resting CD4+ T cells. Nat Med 2003;9:727–8. doi: 10.1038/nm880 12754504 10.1038/nm880

[3] Buzón MJ, Massanella M, Llibre JM, Esteve A, Dahl V, Puertas MC, et al HIV-1 replication and immune dynamics are affected by raltegravir intensification of HAART-suppressed subjects. Nat Med 2010;16:460–5. doi: 10.1038/nm.2111 20228817 10.1038/nm.2111

[4] Hatano H, Strain MC, Scherzer R, Bacchetti P, Wentworth D, Hoh R, et al Increase in 2-long terminal repeat circles and decrease in D-dimer after raltegravir intensification in patients with treated HIV Infection: A randomized, placebo-controlled trial. J Infect Dis 2013;208:1436–42. doi: 10.1093/infdis/jit453 23975885 10.1093/infdis/jit453 PMC3789577

[5] Lorenzo-Redondo R, Fryer HR, Bedford T, Kim E-Y, Archer J, Kosakovsky Pond SL, et al Persistent HIV-1 replication maintains the tissue reservoir during therapy. Nature 2016;530:51–6. doi: 10.1038/nature16933 26814962 10.1038/nature16933 PMC4865637

[6] Fletcher C V, Staskus K, Wietgrefe SW, Rothenberger M, Reilly C, Chipman JG, et al Persistent HIV-1 replication is associated with lower antiretroviral drug concentrations in lymphatic tissues. Proc Natl Acad Sci U S A 2014;111:2307–12. doi: 10.1073/pnas.1318249111 24469825 10.1073/pnas.1318249111 PMC3926074

[7] Pasternak AO, Lukashov V V, Berkhout B. Cell-associated HIV RNA: a dynamic biomarker of viral persistence. Retrovirology 2013;10:41 doi: 10.1186/1742-4690-10-41 23587031 10.1186/1742-4690-10-41 PMC3637491

[8] Shen L, Peterson S, Sedaghat AR, McMahon M a, Callender M, Zhang H, et al Dose-response curve slope sets class-specific limits on inhibitory potential of anti-HIV drugs. Nat Med 2008;14:762–6. doi: 10.1038/nm1777 18552857 10.1038/nm1777 PMC2743464

[9] Kis O, Robillard K, Chan GNY, Bendayan R. The complexities of antiretroviral drug-drug interactions: role of ABC and SLC transporters. Trends Pharmacol Sci 2010;31:22–35. doi: 10.1016/j.tips.2009.10.001 20004485 10.1016/j.tips.2009.10.001

[10] Patterson BK, McCallister S, Schutz M, Siegel JN, Shults K, Flener Z, et al Persistence of intracellular HIV-1 mRNA correlates with HIV-1-specific immune responses in infected subjects on stable HAART. AIDS 2001;15:1635–41. 11546937 10.1097/00002030-200109070-00005

[11] Patterson BK. Simultaneous ultrasensitive subpopulation staining/hybridization in situ (SUSHI) in HIV-1 disease monitoring. Methods Mol Biol 2010;659:337–46. doi: 10.1007/978-1-60761-789-1_26 20809325 10.1007/978-1-60761-789-1_26

[12] Chargin A, Yin F, Song M, Subramaniam S, Knutson G, Patterson BK. Identification and characterization of HIV-1 Latent viral reservoirs in peripheral blood. J Clin Microbiol 2015;53:60–5. doi: 10.1128/JCM.02539-14 25339403 10.1128/JCM.02539-14 PMC4290926

[13] Patterson BK, Mosiman VL, Cantarero L, Furtado M, Bhattacharya M, Goolsby C. Detection of HIV-RNA-positive monocytes in peripheral blood of HIV- positive patients by simultaneous flow cytometric analysis of intracellular HIV RNA and cellular immunophenotype. Cytometry 1998;31:265–74. 9551602 10.1002/(sici)1097-0320(19980401)31:4<265::aid-cyto6>3.0.co;2-i

[14] R Core Team (2016). R: A language and environment for statistical computing. R Foundation for Statistical Computing, Vienna, Austria https://www.R-project.org/.

[15] Sundar Dorai-Raj (2014). binom: Binomial Confidence Intervals For Several Parameterizations. R package version 1.1–1.https://CRAN.R-project.org/package=binom

[16] Sharaf RR, Li JZ. The Alphabet Soup of HIV Reservoir Markers. Curr HIV/AIDS Rep. 2017;14:72–81. doi: 10.1007/s11904-017-0355-y 28401492 10.1007/s11904-017-0355-y PMC5519144

[17] Pasternak AO, Jurriaans S, Bakker M, Berkhout B, Lukashov V V. Steady increase in cellular HIV-1 load during the asymptomatic phase of untreated infection despite stable plasma viremia. Wolters Kluwer Heal AIDS 2010;24:1641–9. 10.1097/QAD.0b013e32833b3171 20543660

[18] Pasternak AO, Jurriaans S, Bakker M, Prins JM, Berkhout B, Lukashov V V. Cellular levels of HIV unspliced RNA from patients on combination antiretroviral therapy with undetectable plasma viremia predict the therapy outcome. PLoS One 2009;4. 10.1371/journal.pone.0008490 PMC2795168 20046870

[19] Hatano H, Jain V, Hunt PW, Lee T-H, Sinclair E, Do TD, et al Cell-based measures of viral persistence are associated with immune activation and programmed cell death protein 1 (PD-1)-expressing CD4+ T cells. J Infect Dis 2013;208:50–6. doi: 10.1093/infdis/jis630 23089590 10.1093/infdis/jis630 PMC3666131

[20] Li JZ, Etemad B, Ahmed H, Aga E, Bosch RJ, Mellors JW, et al The size of the expressed HIV reservoir predicts timing of viral rebound after treatment interruption. AIDS 2015;30:1–28. 10.1097/QAD.0000000000000953 PMC4840470 26588174

[21] Kearney MF, Wiegand A, Shao W, Coffin JM, Mellors JW, Lederman M, et al Origin of Rebound Plasma HIV Includes Cells with Identical Proviruses That Are Transcriptionally Active before Stopping of Antiretroviral Therapy. J Virol 2016;90:1369–76. 26581989 10.1128/JVI.02139-15 PMC4719635

[22] Imamichi H, Dewar RL, Adelsberger JW, Rehm CA, O’Doherty U, Paxinos EE, et al Defective HIV-1 proviruses produce novel protein-coding RNA species in HIV-infected patients on combination antiretroviral therapy. Proc Natl Acad Sci U S A 2016;113:201609057. 10.1073/pnas.1609057113 PMC4978246 27432972

[23] Althaus CL, Joos B, Perelson AS, Günthard HF. Quantifying the Turnover of Transcriptional Subclasses of HIV-1-Infected Cells. PLoS Comput Biol 2014;10. 10.1371/journal.pcbi.1003871 PMC4207463 25340797

[24] Hanley JA, Lippman-Hand A. If nothing goes wrong, is everything all right? Interpreting zero numerators. JAMA 1983;249:1743–5. 6827763

[25] Pasternak AO, De Bruin M, Jurriaans S, Bakker M, Berkhout B, Prins JM, et al Modest nonadherence to antiretroviral therapy promotes residual HIV-1 replication in the absence of virological rebound in plasma. J Infect Dis 2012;206:1443–52. doi: 10.1093/infdis/jis502 22927449 10.1093/infdis/jis502

[26] Falasca F, Maida P, Montagna C, Antonelli L, d'Ettorre G, Monteleone K, et al Expression of the mRNA levels for MDR1, MRP1, MRP4, and MRP5 in HIV antiretroviral naive patients: follow-up at 48 weeks after the beginning of therapy. J Acquir Immune Defic Syndr. 2011;56:e54–6. doi: 10.1097/QAI.0b013e3181fe4c89 21233632 10.1097/QAI.0b013e3181fe4c89

[27] Alam C, Whyte-Allman SK, Omeragic A, Bendayan R. Role and modulation of drug transporters in HIV-1 therapy. Adv Drug Deliv Rev 2016;103:121–43. doi: 10.1016/j.addr.2016.05.001 27181050 10.1016/j.addr.2016.05.001

[28] Kulpa DA, Chomont N. HIV persistence in the setting of antiretroviral therapy: when, where and how does HIV hide? J Virus Erad 2015;1:59–66. 26448966 10.1016/S2055-6640(20)30490-8 PMC4593515

[29] Rosenblum MD, Way SS, Abbas AK. Regulatory T cell memory. Nat Rev Immunol 2015;16:1–12. 26688349 10.1038/nri.2015.1 PMC5113825

